# Climate change stress alleviation through nature based solutions: A global perspective

**DOI:** 10.3389/fpls.2022.1007222

**Published:** 2022-09-21

**Authors:** Muhammad Adil, Zijie Yao, Cheng Zhang, Siqi Lu, Shenglei Fu, Walid F. A. Mosa, Mohamed E. Hasan, Heli Lu

**Affiliations:** ^1^College of Geography and Environmental Science/Key Research Institute of Yellow River Civilization and Sustainable Development and Collaborative Innovation Center on Yellow River Civilization of Henan Province, Henan University, Kaifeng, China; ^2^Department of Geography, University of Connecticut, Storrs, CT, United States; ^3^Key Laboratory of Geospatial Technology for the Middle and Lower Yellow River Regions (Henan University), Ministry of Education/National Demonstration Center for Environment and Planning, Henan University, Kaifeng, China; ^4^Henan Dabieshan National Field Observation and Research Station of Forest Ecosystem, Henan University, Kaifeng, China; ^5^Plant Production Department (Horticulture-Pomology), Faculty of Agriculture, Saba Basha, Alexandria University, Alexandria, Egypt; ^6^Bioinformatics Department, Genetic Engineering and Biotechnology Research Institute, University of Sadat City, Sadat City, Egypt; ^7^Henan Key Laboratory of Earth System Observation and Modeling, Henan University, Kaifeng, China

**Keywords:** climate change, food security, conservation tillage, soil health, nature based solutions

## Abstract

Global climate change stress has greatly influenced agricultural crop production which leads to the global problems such as food security. To cope with global climate change, nature based solutions (NBS) are desirable because these lead to improve our environment. Environmental stresses such as drought and salinity are big soil problems and can be eradicated by increasing soil organic matter which is directly related to soil organic carbon (SOC). SOC is one of the key components of the worldwide carbon (C) cycle. Different types of land use patterns have shown significant impacts on SOC stocks. However, their effects on the various SOC fractions are not well-understood at the global level which make it difficult to predict how SOC changes over time. We aim to investigate changes in various SOC fractions, including mineral associated organic carbon (MAOC), mineral associated organic matter (MAOM), soil organic carbon (SOC), easily oxidized organic carbon (EOC), microbial biomass carbon (MBC) and particulate organic carbon (POC) under various types of land use patterns (NBS), including cropping pattern, residue management, conservation tillages such as no tillage (NT) and reduced tillage (RT) using data from 97 studies on a global scale. The results showed that NT overall increased MAOC, MAOM, SOC, MBC, EOC and POC by 16.2%, 26.8%, 24.1%, 16.2%, 27.9% and 33.2% (*P* < 0.05) compared to CT. No tillage with residue retention (NTR) increased MAOC, MAOM, SOC, MBC, EOC and POC by 38.0%, 29.9%, 47.5%, 33.1%, 35.7% and 49.0%, respectively, compared to CT (*P* < 0.05). RT overall increased MAOC, MAOM, SOC, MBC, EOC and POC by 36.8%, 14.1%, 25.8%, 25.9, 18.7% and 16.6% (*P* < 0.05) compared to CT. Reduced tillage with residue retention (RTR) increased MAOM, SOC and POC by 14.2%, 36.2% and 30.7%, respectively, compared to CT (*P* < 0.05). Multiple cropping increased MAOC, MBC and EOC by 14.1%, 39.8% and 21.5%, respectively, compared to mono cropping (*P* < 0.05). The response ratios of SOC fractions (MAOC, MAOM, SOC, MBC, EOC and POC) under NT and RT were mostly influenced by NBS such as residue management, cropping pattern along with soil depth, mean annual precipitation, mean annual temperature and soil texture. Our findings imply that when assessing the effects of conservation tillage methods on SOC sequestration, SOC fractions especially those taking part in driving soil biological activities, should be taken into account rather than total SOC. We conclude that conservation tillages under multiple cropping systems and with retention of crop residues enhance soil carbon sequestration as compared to CT in varying edaphic and climatic conditions of the world.

## Introduction

The top three meters of world soil is believed to contain 2,344 billion tons of soil organic carbon (SOC), making it the biggest repository of terrestrial organic carbon (Jobbagy and Jackson, 2000). Since soil stores more carbon compared to plants and the environment combined, increasing SOC sequestration is one potential technique to prevent global warming caused by increased carbon dioxide emissions (Schlesinger, 1977; Rumpel et al., 2018). Since SOC directly affects the physical, chemical and biological properties of soil, it is crucial for increasing soil fertility and maintaining soil productivity (Lal, 2004; Rumpel et al., 2018). Considering these facts, SOC might serve as a substitute for evaluating the effects of various managerial and environmental factors on soil services.

However, due to high background SOC concentrations (Zhao et al., 2016) and the presence of recalcitrant C (non-labile C), variations in the SOC fractions are challenging to evaluate (Stockmann et al., 2013). The SOC, on the other hand, responds to management practices more quickly and can be used to measure the effects of management in a shorter amount of time (Liu et al., 2014b; Li et al., 2019b; Kim et al., 2020). They are also thought to be crucial for a variety of soil processes related to production and environmental toughness (Haynes, 2005). Mineral associated organic carbon (MAOC), microbial biomass carbon (MBC), dissolved organic carbon (DOC) and particulate organic carbon (POC) are typical constituents of SOC.

Particularly, DOC is believed to be the key source of energy for soil microbial activity and a sign of the availability of C to soil microbes (Kalbitz et al., 2000). Although it only makes up a minor portion of soil organic carbon (SOC), microbial biomass C has a big impact on various activities performed by microbes (Joergensen and Wichern, 2018). Particulate organic carbon, which is defined as organic C with a particle size ranging from 0.053 to 2 mm, has grown in popularity as a measure for labile SOM estimate due to its ease of quantification (Cambardella and Elliott, 1992). The oxidizable fraction of organic C is easily oxidizable organic carbon, while MAOC is associated with fine soil fractions (silt and clay having diameter of 0.053 mm) and mostly contain compounds of low molecular weight created from microbial and plant activities (Blair et al., 1995; Lavallee et al., 2020). Because these collectively represent a significant group of C fractions that reflect the complex dynamics and important processes in the soil, it has been proposed that these common SOC fractions serve as more sensitive measures for evaluating the effects of nature based solutions (NBS) such as changes in agricultural management practices e.g., conservation tillages.

As an alternate to intensive cultivation (conventional tillage), NBS such as cropping patterns and residue management under conservation tillage practices have been widely adopted in a variety of production systems and have gained popularity as research topics in soil science and agriculture on a global scale.

Numerous studies have indicated that different conservation tillage methods affetcs the typical SOC fractions (Chen et al., 2009; Tivet et al., 2013; Liu et al., 2014b; Somasundaram et al., 2017; Sarker et al., 2018; Bongiorno et al., 2019; Gao et al., 2019). Similarly, based on 10 European long-term field trials, Bongiorno et al. (2019) found that reduced tillage (RT) radically increased concentrations of EOC and POC. Chen et al. (2009) conducted a long term experiment on the Chinese Loess Plateau and found that the proportions of SOC under NT greatly increased in comparison to CT, and sensitivity followed the order EOC > POC > DOC > MBC.

During different experiments conducted in the southern hemisphere, the impact of 47 years of NT on SOC and the related C distribution was examined. The results indicated that the MAOC was 5–12 times higher than POC in the top 0.3 m of soil, and SOC concentrations and stocks were considerably higher under conservation tillages than under CT (Somasundaram et al., 2017).

After 23 years of CT in Brazil's tropical regions, the rate of loss of SOC fractions in the 0–0.2 m soil layer was 0.25 and 0.34 Mg C ha^−1^ year^−1^ for POC and MAOC, respectively (Tivet et al., 2013). In contrast, NT practices increased POC and MAOC concentrations by 0.23 to 0.36 and 0.50 to 0.70 Mg C ha-1 year-1, respectively, in a 8 years experiment (Tivet et al., 2013). The effects of different tillage methods on SOC percent has been extensively studied.

To our knowledge, no global meta-analysis has been carried out to aggregate results from different cropping systems. According to past global assessments, NT without residue retention only had a positive impact on the top 100 mm of the soil (Luo et al., 2010; Mondal et al., 2020). However, double-cropping and NT with residue retention were discovered to be advantageous for SOC in the 0–200 mm range in China after a comprehensive analysis (Du et al., 2017). In light of the published evidence demonstrating that NT is an effective conservation tillage practice, we established to carry out a detailed meta-analysis to determine (1) the effects of conservation tillages, cropping patterns and residue management on the extent and direction of changes in typical fractions of SOC and (2) the effects of different ecological and climatic conditions (e.g., temperature, precipitation and soil texture etc.) in enhancing SOC.

## Materials and methods

**Figure 1:**
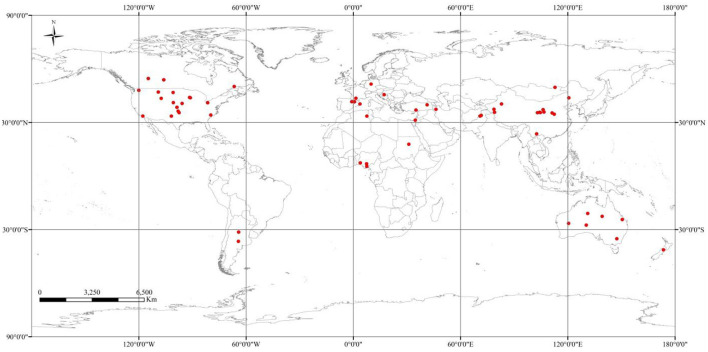
Distribution of 97 experimental sites around the globe from where the data was collected for the meta-analysis.

To find relevant information, we searched peer-reviewed articles using the Web of Science and the Google Scholar by keywords including soil “carbon components,” “conservation tillage techniques” and “cropping patterns.” No tillage with residue retention (NTR) or without residue retention (NTo) and reduced tillage with residue retention (RTR) or without residue retention (RTo) were the specific conservation tillage techniques chosen for comparison in this study. Finally, 97 global papers (1989 to 2021) that satisfied the selection criteria were used in this meta-analysis (Figure 1). The information was manually gathered either from tables and texts in published papers or indirectly from figures by the Get-Data Graph Digitizer software (ver. 2.24, Russian Federation), which was then carefully verified. The final dataset had 283 values for MAOC, 223 values for MAOM, 231 values for SOC, 268 values for MBC, 163 values for EOC, and 219 values for POC concentration.

### Meta-analysis

The data were homogenized into groups based on tillage and cropping patterns. If the study did not contain information on latitude and longitude, soil characteristics, or climatic conditions, this information was looked up online using the following search engine (https://www.whatsmygps.com). The GetData graph digitizer 2.20 program (http://getdata-graph-digitizer.com/index.php) was used to extract data from the figures. The chosen studies' mean annual temperatures (MAT) and mean annual precipitation (MAP) ranged from 3 to 23 °C and 131 to 880 mm, respectively, while the soil textures ranged from sandy loam to clay. We estimated the standard deviation (SD) value using the following formula:


(1) $$\begin{array}{l} SD=SE\;\times \;\sqrt{n} \end{array}$$

Where “n” represents the quantity of samples.

The reciprocal of the variance (V) considered as the weight (W) for each RR was calculated by the following formula (Lucas et al., 2011):


(2) $$\begin{array}{l} W=\;\frac{1}{V} \end{array}$$

Studies having more variance were given less weight in analysis compared to studies having less variance, according to a method suggested by Hedges et al. (1999).

The following formula was used to get the overall mean response ratio (RRE++) for both conventional and conservation tillage:


(3) $$\begin{array}{l} RR_{E++}=\frac{\sum_{i=1}^{n}\sum_{j=1}^{m}W_{ij}RR_{ij}}{\sum_{i=1}^{n}\sum_{j=1}^{m}W_{ij}} \end{array}$$

The letters “n” and “m” stand for the number of treatments and comparisons, respectively, within each category. RR_E_++'s standard error was determined as follows:


(4) $$\begin{array}{l} SE\left(RR_{E++}\right)=\sqrt{\frac{1}{\sum_{i=1}^{n}\sum_{j=1}^{m}W_{ij}}} \end{array}$$

The mean effect size of bias-based bootstrap at 95% confidence interval was calculated using the random model MetaWin 2.1 (Sinaure Associate Inc., Sunderland, USA) to investigate the effects of conservation tillage methods on soil and field parameters (MAOC, MAOM, SOC, MBC, EOC and POC). Using the single observational approach of the Origin 2018, the impact of the conservation tillage practices was considered significant if the 95 % confidence interval did not cross the zero line. Regression analysis of RR to mean annual temperature (MAT) and mean annual precipitation (MAP) was conducted to determine how conservation tillage affects field characteristics as a result of differences in soil and climatic circumstances (OriginLab Corporation, USA).

Meta-analysis was conducted for each categorical sub group such as mean annual precipitation (dry <400 mm; normal, 400–600 mm and wet >600 mm), temperature regions (frigid <8°C; mesic, 8–15°C and thermic > 15°C), and soil texture (fine, medium and coarse) (Dlamini et al., 2016). Soil sampling depth was categorized into topsoil (0–150 mm) and subsoil (150–400 mm), which made up to 68% and 32% of the data groups used in this study, respectively.

Using the mean effect size (lnRR) and its 95% confidence interval, we performed a random-effects meta-analysis with bias adjustment (CI). To evaluate if the expected sample error considerably outweighed the heterogeneity among the lnRR of changes in soil parameters with conservation tillage treatments (Q), a Chi-square test was performed. The significance of group heterogeneity was assessed using a randomization analysis (QB) (Adams et al., 1997). To assess the statistical significance of within-group heterogeneity, chi-square testing was also utilized (QW). Using Rosenthal's Fail-Safe number (Rosenthal and Rosnow, 2008), publication bias was evaluated; if this number is >5n + 10 (where n is the number of observations) the result is regarded as a reliable estimate of the genuine effect (Toth and Pavia, 2007).

## Results

### Effects of NT on MAOC and MAOM

**Figure 2:**
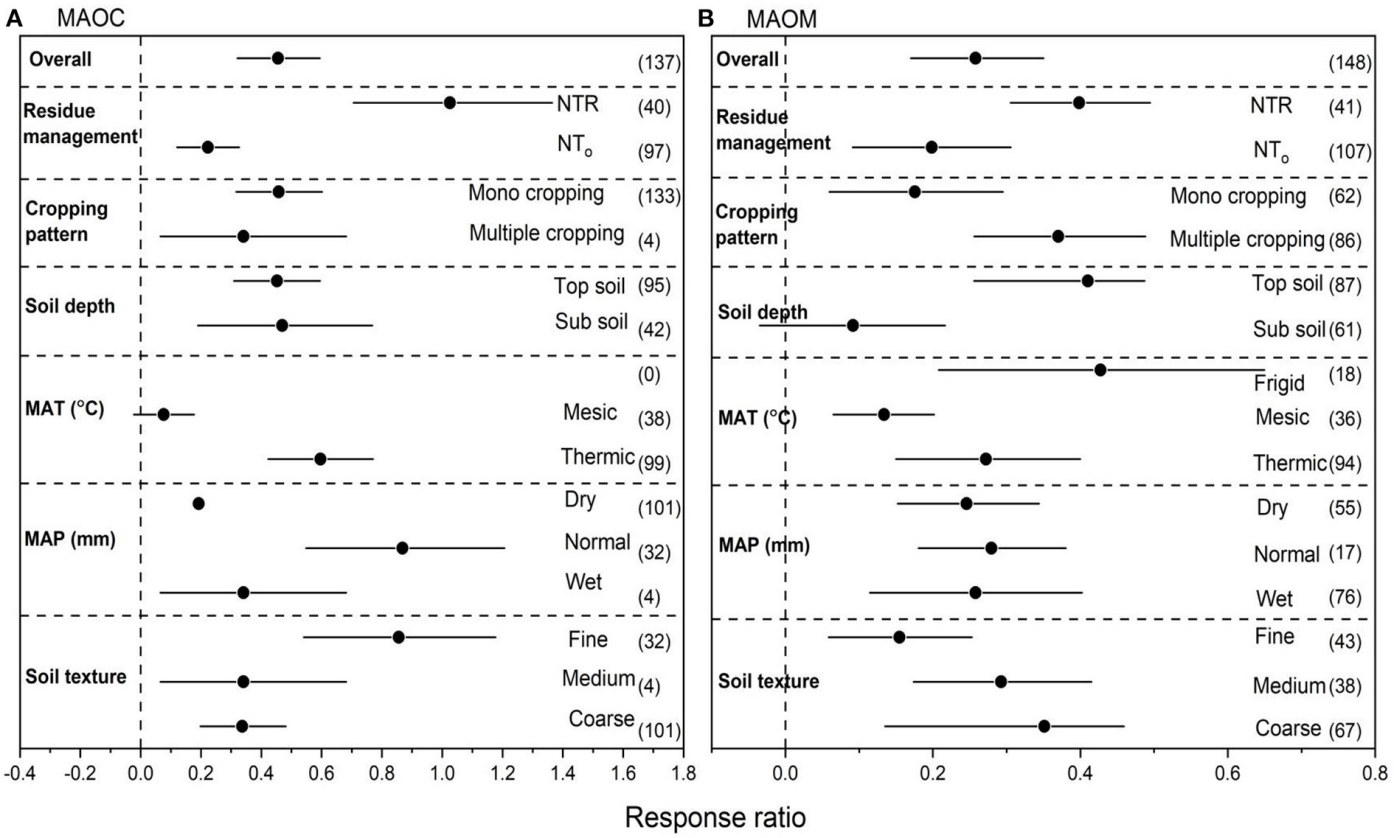
**(A,B)** The effect size of mineral associated organic carbon (MAOC) and mineral associated organic matter (MAOM) with NT compared to CT. If 95% confidence intervals (CIs) do not overlap with zero, there is a substantial difference in the effect size between treatments. Error bars display CIs. The sample size for each variable is displayed next to each bar. The acronyms stand for mean annual temperature (MAT), mean annual precipitation (MAP), retention of residue (NTR), and no residue retention (NTo).

We collected 137 paired observations for MAOC and 148 for MAOM (Figures 2A,B). Data exhibited high heterogeneities as indicated by high Qt values of 127 and 144 for MAOC and MAOM, respectively. NT overall increased MAOC by 26.8% (*P* < 0.05) compared to CT (Figure 2A). Strong interactions were found between NT and crop residues. Among overall management practices under NT, the highest increase in MAOC was observed with NTR (38.0%), followed multiple cropping (29.7%) and mono cropping (27.5%) (*P* < 0.05). Sub soil had greater positive RR for MAOC compared to top soil. The RR of MAOC was greater positive under thermic compared to mesic MAT. Similarly, normal MAP showed greater RR of MAOC compared to dry and wet MAP. The RR of MAOC to overall management practices under NT compared to CT was significant with soil textures (Figure 2A), however, fine textured soils showed greater positive RR for MAOC.

Compare to CT, NT overall increased MAOM by 24.1% (*P* < 0.05) compared to CT (Figure 2B). Strong interactions for MAOM were found between NT and crop residues. Among overall management practices, the highest increase in MAOM was observed with NTR (29.9.0%), followed by multiple cropping (26.7%) and mono cropping (18.4%) (*P* < 0.05). Top soil had greater positive RR for MAOM compared to sub soil. Frigid MAT showed greater RR of MAOM compared to other temperatures. Normal MAP showed greater RR of MAOM compared to dry and wet MAP. The RR of MAOM to overall management practices under NT compared to CT was significant with soil textures (Figure 2B), however, coarse textured soils showed greater positive RR for MAOM compared to other soil textures.

### Effects of NT on SOC and MBC

**Figure 3:**
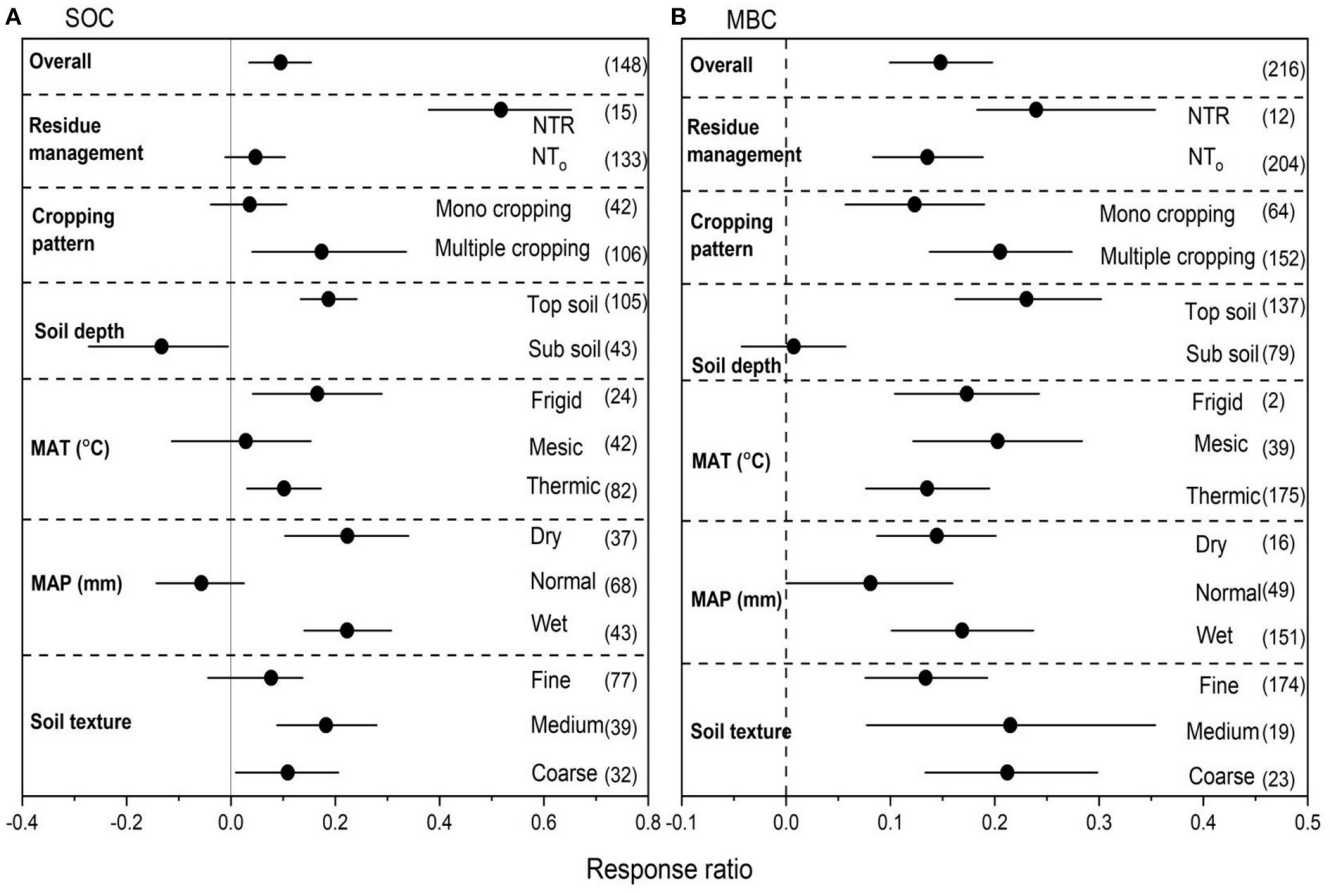
**(A,B)** The effect size of soil organic carbon (SOC) and microbial biomass carbon (MBC) with NT compared to CT. If 95% confidence intervals (CIs) do not overlap with zero, there is a substantial difference in the effect size between treatments. Error bars display CIs. The sample size for each variable is displayed next to each bar. The acronyms stand for mean annual temperature (MAT), mean annual precipitation (MAP), retention of residue (NTR), and no residue retention (NTo).

We collected 148 paired observations for SOC and 216 for MBC (Figures 3A,B). Data exhibited high heterogeneities as indicated by high Qt values of 141 and 197 for SOC and MBC, respectively. NT overall increased SOC by 16.2% (*P* < 0.05) compared to CT (Figure 3A). Strong interactions were found between NT and crop residues. Among overall management practices under NT, the highest increase in SOC was observed with NTR (47.5%), followed by multiple cropping (21.1%) (*P* < 0.05). Top soil had greater positive RR for SOC. However, subsoil had negative RR for SOC under NT. The RR of SOC was greater positive under frigid compared to other temperatures. Wet MAP showed greater RR of SOC compared to dry and normal MAP. The RR of SOC under NT compared to CT was significant with medium and coarse textured soils (Figure 3A), however, medium textured soils showed greater positive RR for SOC compared to coarse-textured soils. NT overall increased MBC by 27.9% (*P* < 0.05) compared to CT (Figure 3B). Strong interactions for MBC were found between NT and crop residues. Among overall management practices, the highest increase in MBC was observed with NTR (33.1%), followed by multiple cropping (28.78%), and mono cropping (20.6%) (*P* < 0.05). Top soil had greater positive RR for MBC compared to sub soil. Mesic MAT showed greater RR of MBC compared to other temperatures. Wet MAP showed greater RR of MBC compared to dry and normal MAP. The RR of MBC to overall management practices under NT compared to CT was significant with soil textures (Figure 3B), however, medium-textured soils showed greater positive RR for MBC compared to other soil textures.

### Effects of NT on EOC and POC

**Figure 4:**
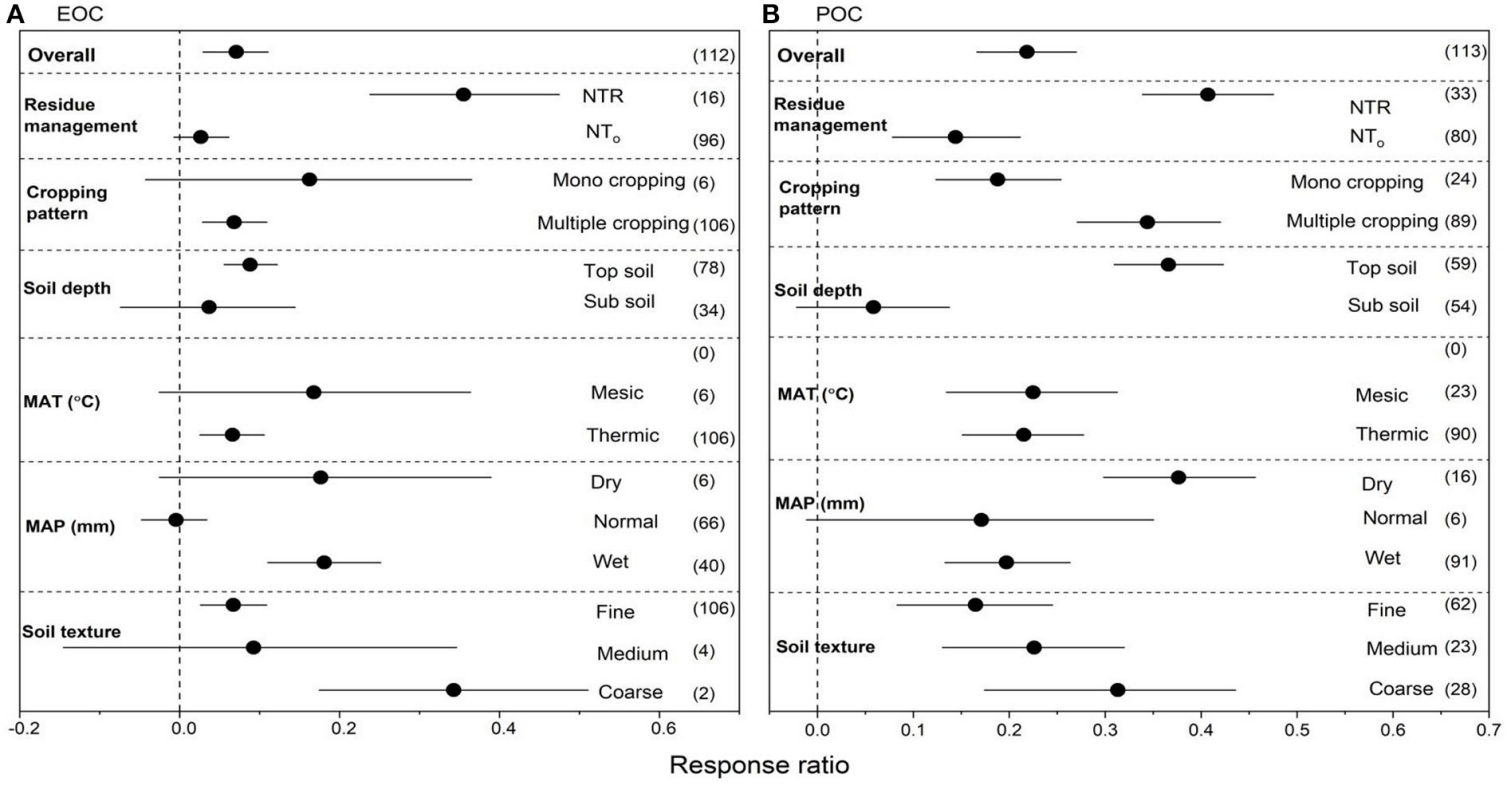
**(A,B)** The effect size of easily oxidized organic carbon (EOC) and particulate organic matter (POC) with NT compared to CT. If 95% confidence intervals (CIs) do not overlap with zero, there is a substantial difference in the effect size between treatments. Error bars display CIs. The sample size for each variable is displayed next to each bar. The acronyms stand for mean annual temperature (MAT), mean annual precipitation (MAP), retention of residue (NTR), and no residue retention (NTo).

We collected 112 paired observations for EOC and 113 for POC (Figures 4A,B). Data exhibited high heterogeneities as indicated by high Qt values of 107 and 110 for EOC and POC, respectively. NT overall increased EOC by 18.7% (*P* < 0.05) compared to CT (Figure 4A). Strong interactions were found among NT, crop residues and multiple cropping. Among overall management practices under NT, the highest increase in EOC was observed with NTR (35.7%), followed by multiple cropping (11.5%) (*P* < 0.05). Mono cropping also increased EOC but not significantly. Top soil had greater positive RR for EOC compared to sub soil. The RR of EOC was greater but not significant under mesic MAT, however, thermic MAT had positive but shorter RR compared to mesic MAT. Wet MAP showed greater RR of EOC compared to dry and normal MAP. The RR of EOC to NT compared to CT was significant in fine and coarse-textured soils but non-significant in medium-textured soils. Compare to CT, NT overall increased POC by 33.2% (*P* < 0.05) compared to CT (Figure 4B). Strong interactions for POC were found between NT crop residues and cropping patterns. Among overall management practices, the highest increase in POC was observed with NTR (49.0%), followed by multiple cropping (38.9%), mono cropping (31.0%) and (25.25) (*P* < 0.05). Top soil had greater positive RR for POC compared to sub soil. Mesic and thermic MAT showed almost similar RR for POC. Dry MAP showed greater positive RR of POC compared to wet MAP while normal MAP showed non-significant RR on POC. The RR of POC to overall management practices under NT compared to CT was significant with soil textures (Figure 4B), however, coarse textured soils showed greater positive RR for POC compared to other soil textures.

### Effects of RT on MAOC and MAOM

**Figure 5:**
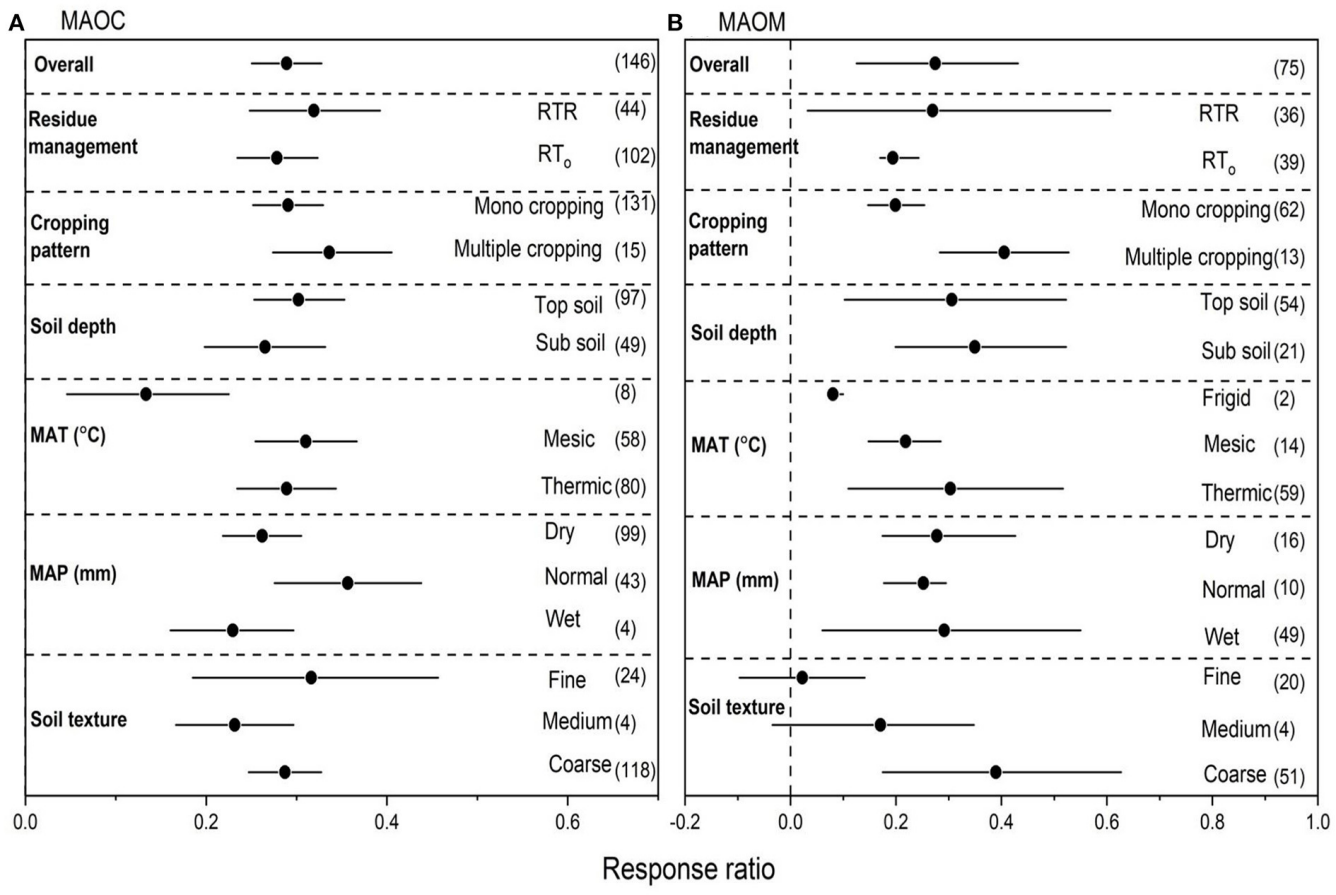
**(A,B)** The effect size of mineral associated organic carbon (MAOC) and mineral associated organic matter (MAOM) with RT compared to CT. If 95% confidence intervals (CIs) do not overlap with zero, there is a substantial difference in the effect size between treatments. Error bars display CIs. The sample size for each variable is displayed next to each bar. The acronyms stand for mean annual temperature (MAT), mean annual precipitation (MAP), retention of residue (RTR), and no residue retention (RTo).

We collected 146 paired observations for MAOC and 75 for MAOM (Figures 5A,B). Data exhibited high heterogeneities as indicated by high Qt values of 139 and 68 for MAOC and MAOM, respectively. RT overall increased MAOC by 36.8% (*P* < 0.05) compared to CT (Figure 5A). Strong interactions were found between RT and crop residues. Among overall management practices under NT, the highest increase in MAOC was observed with multiple cropping (42.5%), followed by RTR (41.1%), and mono cropping (36.3%) (*P* < 0.05). Top soil had greater positive RR for MAOC compared to sub soil. The RR of MAOC was greater positive under mesic compared to other temperatures. Normal MAP showed greater RR of MAOC compared to dry and wet MAP. The RR of MAOC to overall management practices under RT compared to CT was significant with soil textures (Figure 5A), however, fine textured soils showed greater positive RR for MAOC. Compared to CT, RT overall increased MAOM by 14.1% (*P* < 0.05) compared to CT (Figure 5B). Strong interactions for MAOM were found between RT and crop residues. Among overall management practices, the highest increase in MAOM was observed with RTR (14.2%) followed by RTo (12.1%) and multiple cropping (11.7%) (*P* < 0.05). Sub soil had greater positive RR for MAOM compared to top soil. Thermic MAT showed greater RR of MAOM compared to other temperatures. Wet MAP showed greater RR of MAOM compared to dry and normal MAP. The RR of MAOM to overall management practices under RT compared to CT was significant with coarse-textured soils (Figure 5B).

### Effects of RT on SOC and MBC

**Figure 6:**
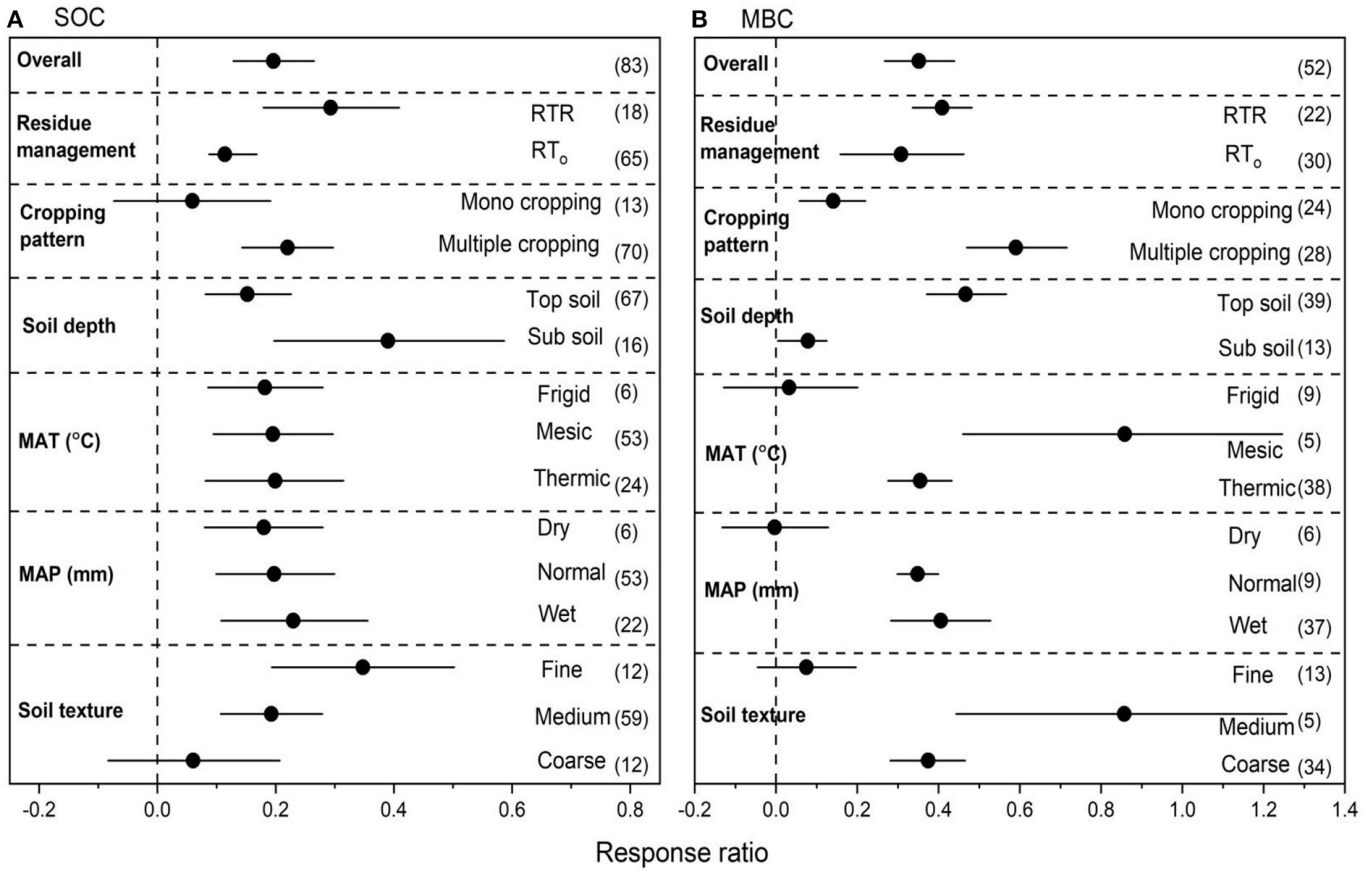
**(A,B)** The effect size of soil organic carbon (SOC) and microbial biomass carbon (MBC) with RT compared to CT. If 95% confidence intervals (CIs) do not overlap with zero, there is a substantial difference in the effect size between treatments. Error bars display CIs. The sample size for each variable is displayed next to each bar. The acronyms stand for mean annual temperature (MAT), mean annual precipitation (MAP), retention of residue (RTR), and no residue retention (RTo).

We collected 83 paired observations for SOC and 52 for MBC (Figures 6A,B). Data exhibited high heterogeneities as indicated by high Qt values of 79 and 50 for SOC and MBC, respectively. RT overall increased SOC by 25.8% (*P* < 0.05) compared to CT (Figure 6A). Strong interactions with found between RT and crop residues. Among overall management practices under NT, the highest increase in SOC was observed with NTR (36.2%), followed by multiple cropping (27.1%) (*P* < 0.05). Sub soil had greater positive RR for SOC. The RR of SOC was greater positive under thermic compared to other temperatures. Wet MAP showed greater RR of SOC compared to dry and normal MAP. The RR of SOC under RT compared to CT was significant with fine and medium-textured soils (Figure 6A), however, fine textured soils showed greater positive RR for SOC compared to coarse-textured soils. Compare to CT, RT overall increased MBC by 25.9% (*P* < 0.05) compared to CT (Figure 6B). Strong interactions for MBC were found between RT and crop residues. Among overall management practices, the highest increase in MBC was observed with multiple cropping (39.8%) followed by NTR (28.9%) (*P* < 0.05). Top soil had greater positive RR for MBC compared to sub soil. Mesic MAT showed greater RR of MBC compared to other temperatures. Wet MAP showed greater RR of MBC compared normal MAP. However, dry MAP did not show positive RR for RT. The RR of MBC under RT compared to CT was significant with medium and coarse-textured soils (Figure 6B), however, fine textured soils did not show significant RR for SOC.

### Effects of RT on EOC and POC

**Figure 7:**
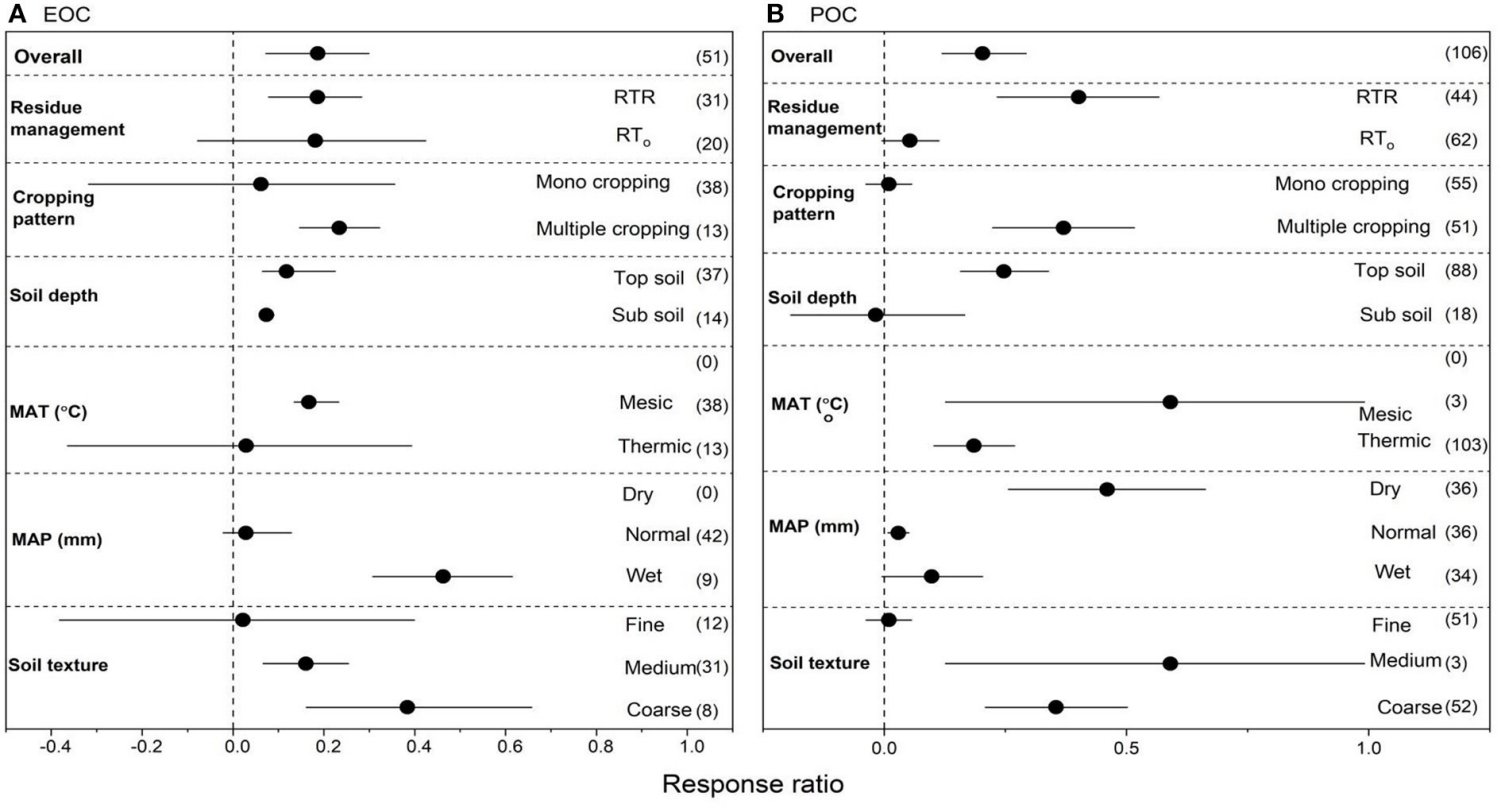
**(A,B)** The effect size of easily oxidized organic carbon (EOC) and particulate organic matter (POC) with RT compared to CT. If 95% confidence intervals (CIs) do not overlap with zero, there is a substantial difference in the effect size between treatments. Error bars display CIs. The sample size for each variable is displayed next to each bar. The acronyms stand for mean annual temperature (MAT), mean annual precipitation (MAP), retention of residue (RTR), and no residue retention (RTo).

We collected 51 paired observations for EOC and 106 for POC (Figures 7A,B). Data exhibited high heterogeneities as indicated by high Qt values of 50 and 102 for EOC and POC, respectively. RT overall increased EOC by 18.7% (*P* < 0.05) compared to CT (Figure 7A). Strong interactions were found between RT, crop residues and mono cropping. Among overall management practices under RT, the highest increase in EOC was observed with multiple cropping (21.5%) followed by RTR (18.5%) (*P* < 0.05). Mono cropping also increased EOC but not significantly. Top soil had greater positive RR for EOC compared to sub soil. The RR of EOC was greater but not significant under thermic MAT, however, mesic MAT had positive but shorter RR compared to thermic MAT. Wet MAP showed positive RR of EOC compared to dry and normal MAP. The RR of EOC to RT compared to CT was significant with coarse and medium-textured soils but non-significant with fine-textured soils. Compare to CT, RT overall increased POC by 16.6% (*P* < 0.05) compared to CT (Figure 7B). Strong interactions for POC were found between RT crop residues and cropping patterns. Among overall management practices, the highest increase in POC was observed with RTR (30.7%), followed by multiple cropping (27.1%) (*P* < 0.05). However, mono cropping and RTo had positive RR but not significant. Top soil had greater positive RR for POC compared to sub soil. Mesic MAT showed greater positive RR for POC. Dry MAP showed greater positive RR of POC compared to wet MAP while normal MAP showed non-significant RR on POC. The RR of POC to overall management practices under RT compared to CT was significant with medium and coarse-textured soils while non-significant for fine-textured soils.

### Correlations of MAOC, MAOM and MBC to SOC

**Figure 8:**
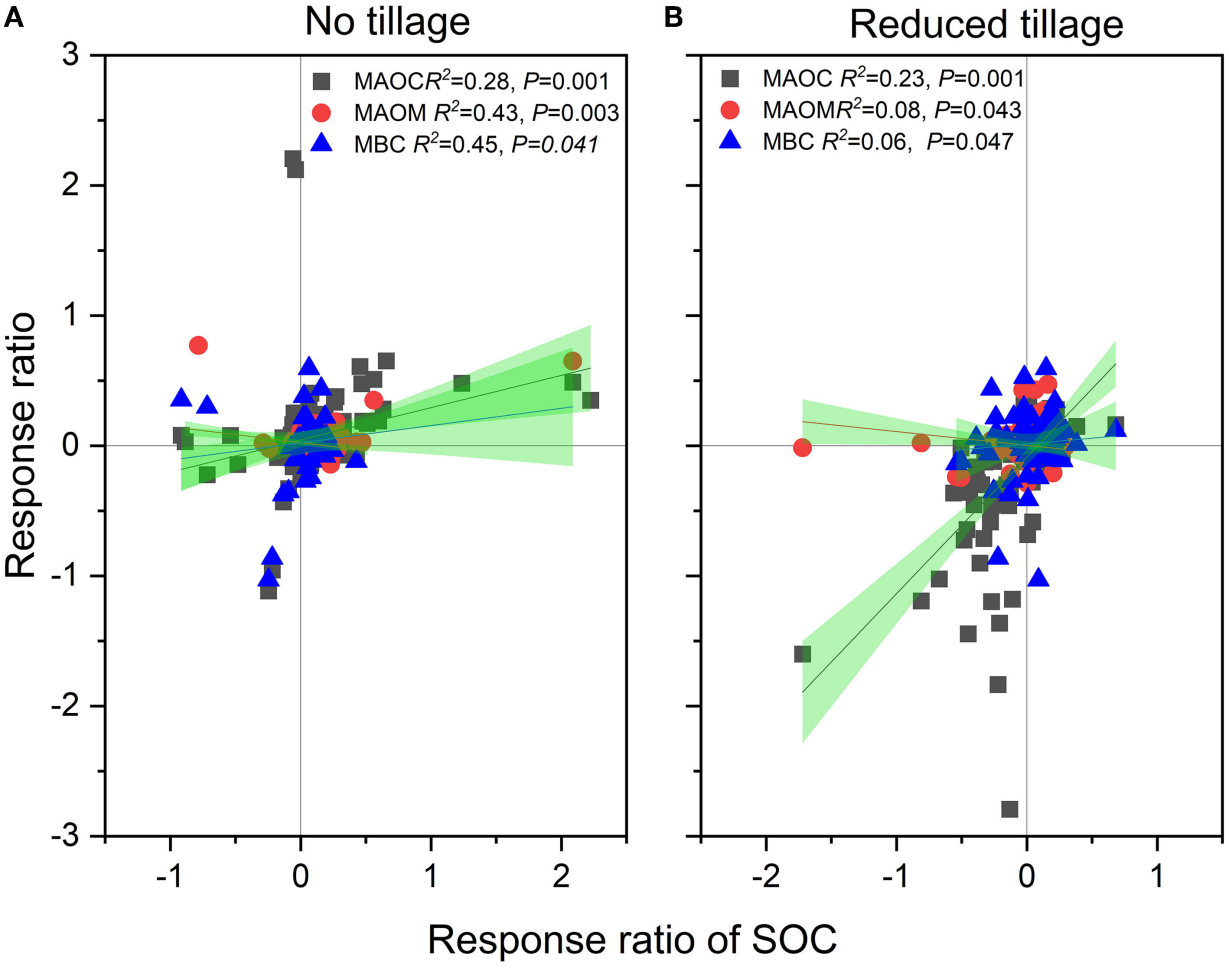
**(A,B)** Correlations of the response ratio of MAOC, MAOM and MBC to that of SOC.

The RR of SOC for NT and RT increased linearly with the RR of MAOC (Figure 8). The rise in the RR of SOC for NT and RT practices can be attributed to 16.2 and 36.8% increase in the RR of MAOC, respectively. The RR of MAOM was also connected to the RR of SOC for NT and RT practices, such as a rise in the RR of SOC for conservation tillages can be attributed to 26.8% and 14.1% increase in the RR of MAOM, respectively.

Similarly, the RR of MBC was related to that of SOC such as the rise in RR of MBC for NT and RT practices can be used to explain 16.2% and 25.9% increase in MBC under NT and RT practices, respectively.

**Figure 9:**
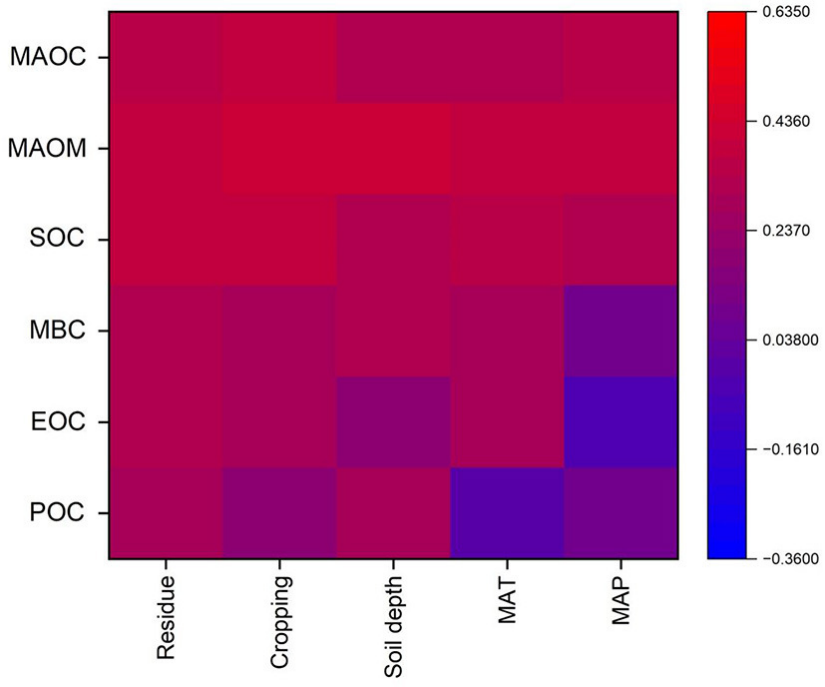
The Pearson correlations between the response ratio (conservation tillages (NT, RT) vs. conventional tillage) of soil fractions including mineral-associated organic carbon (MAOC) and mineral-associated organic matter (MAOM), soil organic carbon (SOC), microbial biomass C (MBC), easily oxidized organic carbon (EOC) and particulate organic carbon (POC) under different types of land use (Cropping pattern, residue management, CT, NT and RT) and climatic factors such as MAP and MAT. Correlation coefficients have designated by the color gradients.

The Pearson correlations between the response ratio (conservation tillages (NT, RT) vs. conventional tillage) of soil fractions including mineral-associated organic carbon (MAOC) and mineral-associated organic matter (MAOM), soil organic carbon (SOC), microbial biomass C (MBC), easily oxidized organic carbon (EOC) and particulate organic carbon (POC) under different types of land use (cropping pattern, residue management, CT, NT and RT) and climatic factors such as MAP and MAT has shown in Figure 9.

## Discussions

### Responses of SOC sequestration under different management practices

In context of global climate change, increasing soil C sequestration through climate-smart agricultural practices has achieved a lot of interest. This consensus among the general public and the scientific community is reflected in a sizable body of literature on residue management (Liu et al., 2014a) and NT application (Luo et al., 2006), or both combined at the regional and global scales (Mondal et al., 2020). Previous research suggests that specific SOC fractions, as opposed to the entire SOC pool probably have a greater influence on soil microbial community, nutrient cycling and availability, and aggregation, making them a more accurate and trustworthy indicator of changes carried by management practices and environmental factors. POC (Cui et al., 2014) and MBC (Li et al., 2019a) are two soil C fractionation approaches that have been successfully introduced and verified using field data (Culman et al., 2012). Each SOC fraction metric's utility and sensitivity remain poorly understood, and it is unclear how different climatic, edaphic and field management conditions might interact with or affect these variables. By thoroughly investigating all key SOC fractions impacted by conservation tillage options using a global meta-analysis approach, we aimed to fill in these information gaps. Each average SOC percentage generally matched the overall SOC, and conservation tillage techniques resulted in higher SOC concentrations than conventional tillage techniques.

The RR of SOC was closely correlated with the RRs of several SOC fractions, including MAOC, MAOM, MBC, EOC, and POC (Figures 2–7) that supports past findings (Chen et al., 2009; Orgill et al., 2017). Whereas, these common fractions were significantly impacted by the agronomic and environmental factors that affected overall SOC concentrations (Figures 2–7). We were able to pinpoint variations among the numerous factors that influenced the different SOC percentages by fusing the Pearson's correlation analysis and subgroup meta-analysis. The conservation tillages (NT and RT) were consistently in good correlation with MAOC, MAOM, MBC, EOC and POC, were the cause of the higher SOC concentrations found in our study. Despite comprising up only 5% of the total amount of soil organic matter, soil MBC is responsible for a greater part of carbon sequestration (Figures 3, 6) than other SOC fractions for two reasons. First, MBC is more vulnerable to environmental and agronomic conditions (Tivet et al., 2013; Liu et al., 2014b; Zhao et al., 2016; Orgill et al., 2017). The current study also found that changes in EOC and POC have only a weak link with SOC changes, while percentage of SOC such as MAOC, MAOM, and MBC have a considerable impact on overall SOC changes (Figure 9). Additionally, soil microbes indirectly affect C cycling by promoting soil aggregation and the ensuing generation of POC (Six et al., 2000).

MBC is a promising component for regulating alterations in the soil C pool in this regard. Second, because it directly controls the activities of soil extracellular enzymes that are crucial for fostering SOC turnover, MBC is crucial for the turnover of different SOC fractions (Joergensen and Wichern, 2018). Since certain bacteria may be latent, high MBC concentrations may not always signify active bacteria. An extensive meta-analysis found that NT increased culturable microbial populations rather than more intricate elements like soil microbial diversity and community structure (Li et al., 2020). Furthermore, the priming effect and the entombing effect, two microbially produced C processes, were employed to regulate the variation of the stable soil C pool (Liang et al., 2017). On the other side, the entombing effect assumes that when microbes produce biomass, they synthesize new organic molecules, in this way some of their necromass are stabilized. The priming effect shows that C losses dramatic increase with the addition of fresh external C by accelerating the microbial breakdown of stabilized soil organic matter. Studies have revealed that microbial-derived C is widely distributed and fairly stable against breakdown when it is physically kept (Liang et al., 2017; Cotrufo et al., 2019).

We suggest that the application of NT/RT will increase SOC in cropland soils because less disturbance and/or residue addition directly or indirectly increased total soil microbial biomass by improving microclimatic and nutrient conditions (Figures 3, 6b). This is supported by the correlations and strength of relationships between the RR of SOC to that of MAOC, MAOM, MBC, EOC, and POC (Figure 9). Once stable forms of carbon (such as POC, that is protected from microbial destruction by aggregation) can be stored for a long period, increased SOC sequestration will be feasible (Figures 4, 7) (Six et al., 2000; Liang et al., 2017; Lavallee et al., 2020). However, more research is needed to back up the suggested procedure.

According to a previous meta-analysis on the effects of NT on SOC (Luo et al., 2010; Mondal et al., 2020), the concentrations of SOC decreased rapidly with increasing soil depth (Figure 3). This could be explained by the accumulation of organic material on the topsoil, which provides substrate sufficiency and a favorable habitat for soil microorganisms. According to Six et al. (2000) the concentrations of MAOC, MAOM, MBC, EOC, and POC in the topsoil under NT were boosted as a result of the increased labile SOC fractions in the topsoil (Figure 3). Our results qualitatively agree with findings from related studies (Liu et al., 2014b; Mondal et al., 2020). Due to decreased nutrient concentrations (Ashagrie et al., 2007), decreased water storage (Resck et al., 2008), and greenhouse gas emissions, the decline in SOC concentration has serious agricultural and environmental consequences because it is a key indicator of soil quality for the production of sustainable crops (Lal, 2004, 2006). In addition, tillage removes soil aggregates that serve as a physical barrier between SOC and microbial degradation (Tisdall and Oades, 1982; Beare et al., 1994). It is crucial to provide farmers with alternative soil management techniques that lessen SOC degradation and improve soil aggregation in dryland farming systems. Our finding suggest that RTR increased SOC concentration with crop residues left behind (Figure 6) and the results followed the previous findings (Halvorson et al., 2002).

Our finding suggest that NTR increased SOC concentration with crop residues left behind (Figure 3) and the results followed the previous findings such as NTR slower the rate of residue decomposition that may cause more SOC to accumulate in the topsoil (Figure 3) (Álvaro-Fuentes et al., 2008). Numerous investigations have shown that long-term conservation tillage application causes SOC building to be higher in semi-arid environments than when using conventional approaches (Álvaro-Fuentes et al., 2008; Hernanz et al., 2009; López-Fando and Pardo, 2011). Under RTR treatment, there was less physical disintegration of organic matter and more organic matter was present as crop residues (Figure 5), which resulted in accumulation of SOC (Figure 6) and a noticeably higher concentration of MBC (Figure 6).

The availability of organic matter as a source of energy for soil microorganisms has led to a larger level of microbial biomass in surface soil (Wright et al., 2005). Since soil MBC reacts quickly to changes in soil management, it has been hypothesized that it is a more sensitive indicator of changes in soil quality carried by different soil management practices (Filip, 2002; Biederbeck et al., 2005). Retention of residue greatly enhanced POC under RTR compared to RT_o_ in the current research. POC grows as a result of agricultural waste products and wheat residual roots (Yoo and Wander, 2008). The POC percentage in this study ranged from 9.8 to 30.2 % of the total SOC, which is consistent with normal values of 10 to 30 % as described in the literature (Wander, 2004; Álvaro-Fuentes et al., 2008; Lammerding et al., 2011). Nevertheless, in spite of its small size, it significantly affects the soil's capacity to provide nutrients and preserve structural stability, making it an essential element of soil quality (Haynes, 2005). Continuing plowing and diminishing binding agent concentrations may be the cause for the loss in structural stability in CT as compared to NT and RT. Numerous studies found that tillage practices increased the probability of macroaggregates breakdown (Mikha and Rice, 2004; Ashagrie et al., 2007).

### Effects of temperature and soil texture on SOC sequestration

In our study, SOC content was closely connected with MAT (Figures 3, 6), which has previously been proven by numerous studies demonstrating how climatic conditions greatly affected SOC contents (Kalbitz et al., 2000; Hermle et al., 2008; Bongiorno et al., 2019) such as temperature affects SOC turnover rate, plant development in response to heat unit building and microbial activities (Zhao et al., 2016). This may also be the cause of the insignificant variations in the RR of EOC between NT and CT when MAP > 1,000 mm (Figure 4). The lower RR of EOC and POC at frigid (8–15°C MAT) suggests that EOC and POC degrade more quickly than they generate at that temperature, which may be due to a variety of different causes such as fresh C resource inputs into the rapidly rotating SOM may result in residues at relatively low temperatures, trigger biological priming processes that speed up the breakdown of labile C resources (Kuzyakov et al., 2000). More microbial activity also encourages the immobilization of bioavailable C resources, such as EOC (Kalbitz et al., 2000). Both could result in additional EOC and POC losses.

This study showed that the physical elements of SOC, including POC and MAOC can be fairly stable over time. This may have been influenced by the accumulation of SOM, which is a source of POC (Filep and Rékási, 2011). Additionally, clay has a high capacity for holding water and a high affinity for POC adsorption, which minimizes POC leaching (Figures 4, 7) (Don and Schulze, 2008). Soil texture is a significant component that affects soil porosity and the soil's ability to maintain SOM. The essential physicochemical characteristics that are particular to some soil types control how well soils may hold different SOC fractions (Six et al., 2002).

This meta-analysis showed that the medium textured soils increased different C sources compared to fine and coarse textured soils (Figures 2, 3, 6, 7). In general, loamy soil reflects a soil texture that is well-balanced in terms of nutritional condition, microclimate sites, and hydraulic features (Soil Survey Staff, 2014). As a result, the application of NT and RT in this study considerably increased the RR of SOC fractions like MAOM, MAOC, MBC, EOC, and POC under medium textured soils. Conservation tillage strategies enhanced SOC concentrations in medium textured soils, and the total SOC was typically in agreement with each typical SOC fraction (Figures 2–7) (Abdalla et al., 2016).

## Conclusion

Our meta-analysis found that the nature based solutions (NBS) such as non-removal of crop residues and multiple cropping under conservation tillages overall increased SOC concentrations and were mainly correlated with three SOC fractions such as MAOC, MAOM and MBC. Cropping pattern, soil depth, and MAT were the parameters that had the greatest influence on the SOC fractions among the numerous environmental and agronomic factors. The extensive use of these management practices in agricultural research as indicators of changes in soil health is the result of the well-documented sensitivity of various common SOC fractions. The previous few decades have seen extensive use of NT in numerous regions throughout the world. By providing details on the interactions between tillage management and distinct SOC factions, the study's findings contribute to filling in knowledge gaps. In particular, applying NT can potentially be a successful strategy for enhancing SOC pool within the 0–150 mm soil profile in medium-textured soil regions with 1,000 mm MAP and 8-15 °C MAT under multiple cropping systems. The increased SOC and SOM improve soil health and help to eradicate environmental stresses such as salinity and drought. This effect should primarily be attributed to the increased microbial derived organic C fractions.

## Data availability statement

The original contributions presented in the study are included in the article/supplementary material, further inquiries can be directed to the corresponding author/s.

## Author contributions

All authors listed have made a substantial, direct, and intellectual contribution to the work and approved it for publication.

## Funding

This study is under the auspices of NSFC 42071267, the Program for Innovative Research Team (in Science and Technology) with the University of Henan, Henan Province (21IRTSTHN008) and the Scientific and Technological Research Projects in Henan Province (222102320472).

## Conflict of interest

The authors declare that the research was conducted in the absence of any commercial or financial relationships that could be construed as a potential conflict of interest.

## Publisher's note

All claims expressed in this article are solely those of the authors and do not necessarily represent those of their affiliated organizations, or those of the publisher, the editors and the reviewers. Any product that may be evaluated in this article, or claim that may be made by its manufacturer, is not guaranteed or endorsed by the publisher.
